# DNS and Experimental Assessment of Shark-Denticle-Inspired Anisotropic Porous Substrates for Drag Reduction

**DOI:** 10.3390/biomimetics10120838

**Published:** 2025-12-15

**Authors:** Benjamin Kellum Cooper, Sasindu Pinto, Henry Hong, Yang Zhang, Louis Cattafesta, Wen Wu

**Affiliations:** 1 Department of Mechanical Engineering, University of Mississippi, 1764 University Circle, Oxford, MS 38677, USA; bkcoope1@go.olemiss.edu; 2 Department of Mechanical, Materials, and Aerospace Engineering, Illinois Institute of Technology, 10 W 32nd St., Chicago, IL 60616, USA; spinto6@hawk.illinoistech.edu (S.P.); hhong7@illinoistech.edu (H.H.); yzhang215@illinoistech.edu (Y.Z.); lcattafestaiii@illinoistech.edu (L.C.)

**Keywords:** flow control, porous media, drag reduction, shark skin, anisotropic permeability, reverse pore flow, bio-inspired surface morphologies

## Abstract

Passive flow control methods are widely used to reduce drag in wall-bounded flows. A recent numerical study on separating turbulent flows over a bump covered with shark denticles revealed the formation of a reverse pore flow (RPF) beneath the denticle crowns under an adverse pressure gradient (APG). This RPF generates an upstream thrust, leading to drag reduction. Motivated by these findings, the present study investigates a bio-inspired Anisotropic Permeable Propulsive Substrate (APPS) that incorporates key geometric features of the shark denticles, enabling thrust generation by the RPF. The designed APPS is evaluated through both direct numerical simulations of turbulent channel flows at $Re_{\tau }$ = 1500 and experiments using 3D-printed structures in a turbulent boundary layer over a flat-plate model subjected to APG and flow separation (at $Re_{\theta }$ = 800). Both approaches demonstrate that the APPS successfully reproduces the RPF-induced thrust mechanism of shark denticles. The results further reveal the dependence of the pore flow on pressure gradient and substrate geometry. This work highlights two features of a thrust-generating APPS: a top surface that shields the porous media from the overlying flow while enabling vertical mass exchange, and a bottom region with dominant wall-parallel permeability, which guides the pore flow in the streamwise direction to generate the thrust.

## 1. Introduction

Research on efficient flow-control strategies, including those aimed at drag reduction and stall prevention or delay in aerodynamic flows, remains a central focus in fluid dynamics. In particular, controlling flows subjected to adverse pressure gradients (APGs) or even separation—detachment of fluid from a surface—continues to pose significant challenges [1,2]. Because the surfaces of air vehicles are inherently curved—with regions of contraction, expansion, and local protrusions from installed equipment—APGs and flow separation are naturally present and unavoidable. Similar complexities arise in marine applications, where effective control of drag and flow separation could provide substantial benefits for ships and other vessels.

Many flow-control approaches are motivated by nature [3], such as the streamlined body shapes of fish [4,5,6], leading-edge vortex generators that emulate the effect of the alular feathers on spread-wing birds [7,8,9,10], and streamwise-aligned riblets inspired by shark skin [11,12,13,14]. These bio-inspired strategies provide effective control without requiring additional energy input, exemplifying passive flow control (PFC) that modulates the flow via geometric modifications. Recent advances in additive manufacturing have enabled accurate fabrication of complex microsurface structures, offering unprecedented potential for utilizing bio-inspired surface structures.

**Figure 1:**
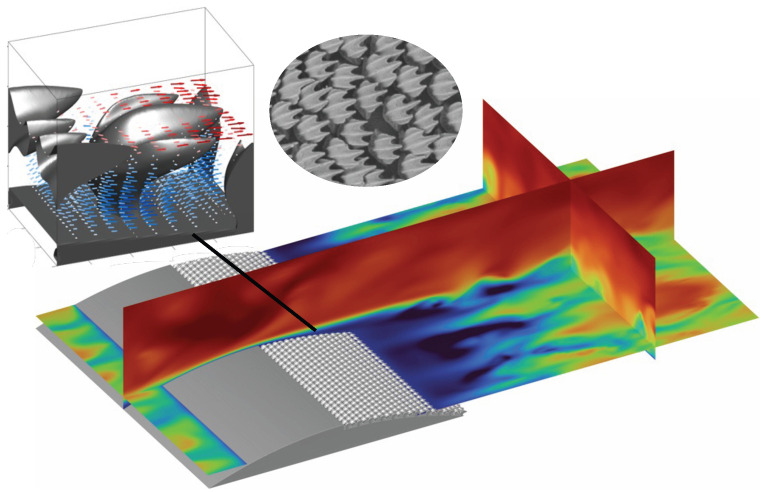
Turbulent channel flow over a bump on one side of the channel wall [31]. The lee side of the bump is covered by shark denticle replicas. The contours in the three two-dimensional slices of the flow field show the instantaneous streamwise velocity, highlighting the flow detachment over the denticle replicas. The inset three-dimensional view shows the velocity vectors around a denticle which exhibits a reverse (upstream) pore flow formed beneath the crown.

While PFC does not require external energy input for actuators, it can incur a drag penalty under off-design operating conditions. For instance, riblets have shown effectiveness only within a narrow speed range [15,16,17,18,19,20] and primarily on the straight sections of aircraft or ship bodies; outside these conditions, their drag-reduction performance deteriorates rapidly and can even become drag-augmenting [21,22,23]. Shark skin, which is covered with tiny dermal scales known as denticles (see inset in Figure 1), is found to increase drag during cruise [24,25,26,27] even though the ridges over their crown are believed to work as riblets to reduce drag. Other studies focus on the bristling of denticles as a mechanism to control flow separation and reduce pressure drag [28,29,30]. A recent study by Savino and Wu [31] proposed a new PFC approach using a porous layer formed by indented shark denticles. By examining the flow between shark denticles under APGs in a turbulent channel flow separating over a bump whose lee side is covered by shark denticle replicas, they discovered a reverse pore flow (RPF) (opposite to the bulk flow, going upstream) beneath each denticle that generates upstream thrust, leading to significant overall drag reduction. Specifically, this drag reduction occurs when there is APG over the shark denticle replicas, before the onset of the massive flow separation. Two geometric features of the denticles are found to be critical for this mechanism: The crown that enables mass exchange between the outer flow and the pore flow, and the staggered necks that channel the RPF. In addition, the backward-facing slits between adjacent denticle crowns are thought to promote the penetration of outer flow into the denticle layer under APGs. This arrangement differs from canonical porous media in that it exhibits two distinct forms of permeability. The top layer only allows the flow to go into/out of the bottom layer, while the bottom neck region confines the flow to be mostly wall-parallel so the thrust is in the upstream direction. While various porous media and permeable surfaces have been studied [32,33,34,35,36], a key advantage of the present two-layer porous structure is that the denticle neck region does not introduce additional drag under ZPG flow while still providing drag reduction under APG conditions.

In this paper, we aim to develop a bio-inspired porous substrate that mimics the geometry of shark denticles while maintaining or exceeding their drag-reduction performance. Our hypothesis is that the thrust-generation functionality of the shark denticle observed in ref. [31] can be reproduced by engineered, manufacturable substrates that do not share the structural limitation of shark denticles—namely, that each denticle must grow and stand individually to form a functional array—but can instead be fabricated as complete, integrated structures. We refer to this new design as the Anisotropic Porous Propulsive Substrate (APPS). The simulations aim to elucidate the thrust-generation mechanisms identified by Savino and Wu [31] using an APPS configuration that avoids the biological constraints of shark denticles while preserving their hydrodynamic advantages. In addition, we employ a novel channel-flow configuration that enables spatial transitions between FPG, ZPG, and APG, allowing us to examine the development of pore flow under all three pressure-gradient conditions (whereas Savino and Wu [31] examined only APG) at reasonable computational costs. The experimental effort focuses on verification of drag reduction via advanced additive manufacturing of miniature denticle-like geometries and the designed APPS, and on characterizing their overall drag-reduction performance using a floating flat-plate model and a force balance in a wind tunnel.

The novelty of this work lies in the abstraction of shark denticles into a simplified, manufacturable porous substrate; the combined use of direct numerical simulations and experimental validation to demonstrate the thrust-generation capability of the newly designed APPS; and the first investigation of the substrate’s response to various pressure gradients.As we show in this paper, the APPS—developed based on the two-layer anisotropic permeability of shark denticles—successfully generates thrust under an adverse pressure gradient. The necessity of incorporating both layers is demonstrated. However, the preferential response to adverse pressure gradients and the corresponding suppression of drag-augmenting forward pore flow was not achieved using the inclined slit identified in ref. [31], indicating that further investigation is warranted.

The APPS design is introduced in Section 2.1, followed by numerical and experimental methodology summarized in Section 2.2, Section 2.3 and Section 2.4. Next, we discuss the instantaneous and mean flow features observed in the simulations in Section 3.1 and Section 3.2, as well as the forces created by the APPS in Section 3.3. Experimental results are discussed in Section 3.4. Section 4 concludes the study by summarizing the findings and offering physical insights and envisioned future work.

## 2. Methodology

### 2.1. APPS Design

**Figure 2:**
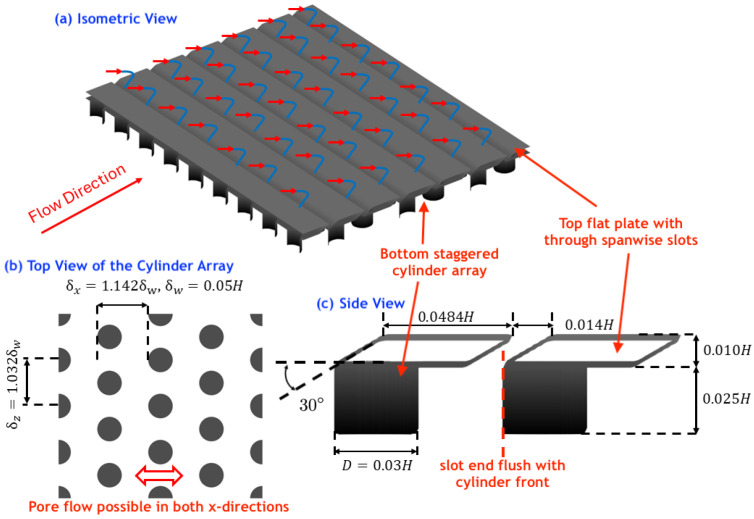
Schematic and parameters of APPS. *H* is the channel half height. (**a**) Isometric view with arrows indicating forward (red) flow over the APPS turns backwards (blue) when entering the backward-facing slots. (**b**) Top view of the cylinder array region staggered arrangement. (**c**) Side view showing the alignment of the slots and the cylinders.

The APPS consists of two layers (Figure 2a). The top layer permits flow penetration, mimicking the one formed by the crowns of shark denticles. The crown ridges are omitted because prior work [31] confirmed that they do not influence the RPF-induced thrust generation; a flat surface is therefore used. Spanwise homogeneous slots are cut into the top plate to represent the backward-facing slits between denticle crowns (Figure 2a,c). The lower region beneath the top plate consists of an array of circular cylinders arranged in a staggered pattern (Figure 2b), analogous to the denticle necks that channel the RPF. On the underside of the top plate, the rear edge of each slot is aligned with the leading edge of the corresponding row of cylinders (Figure 2c). In this study, the total height of the APPS and the spacing of the cylinder arrays are selected based on the values reported for shark denticle arrays in ref. [31]. This design is utilized in both simulations and experiments, in which two different flow configurations are employed to assess its performance.

### 2.2. High-Fidelity Simulations

#### Configuration

Direct numerical simulations (DNS) of turbulent channel flows over various APPS configurations are conducted at friction Reynolds number $Re_{\tau }=U_{\tau }H/\nu =$ 1500, where *H* represents the channel half-height and $U_{\tau }$ the average friction velocity based on the total drag. The simulations are run with a constant pressure gradient, which maintains turbulence at the viscous scale required to resolve interactions with the APPS. In addition to a baseline case representing the canonical zero-pressure-gradient (ZPG) channel, five cases incorporating an insert at the channel centerline that creates PGs along the channel are performed. This configuration allows the flow to transition between FPG, ZPG, and APG over a relatively short streamwise distance, since resolving the pore flow within the APPS—whose height is only a few percent of the boundary-layer thickness or channel half-height—over a long streamwise extent requires billions of grid points and intractable CPU hours. Although it does not represent a specific realistic flow condition, this simplified setup is designed to isolate and examine the influence of different pressure gradients on the flow within the porous media at an acceptable (but still significant) computational cost. The designed APPS, when present, are placed on the top and bottom walls of the channel. Statistics are averaged between the two walls due to symmetry. The top plate of the APPS is set to 0.01*H* thick, and the bottom cylinders are 0.025*H* tall and 0.03H in diameter. The computational domain is about 7.489H in the streamwise direction (*x*), 2H in the wall-normal direction (*y*), and 3.384H in the spanwise direction (*z*), sized to ensure the periodicity of the structures in the streamwise and spanwise directions. The slot angle is set to 30° as the default, matching the inclination of the denticle crown’s bottom surface. The slot width is chosen to be 0.014*H*, resulting in 22.4% of the top plate open area to allow mass exchange above and below.

**Figure 3:**
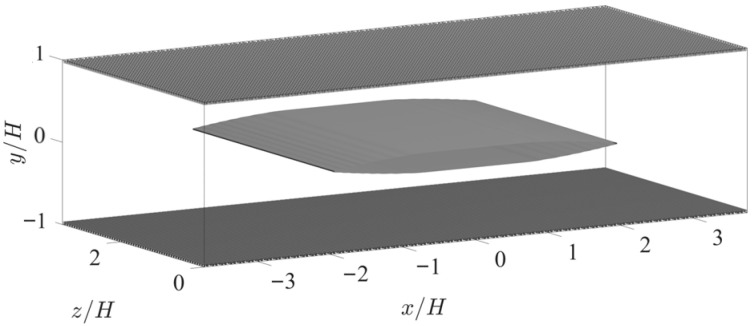
Computational configuration with a symmetric NACA 0024–based insert placed along the channel centerline.

**Figure 4:**
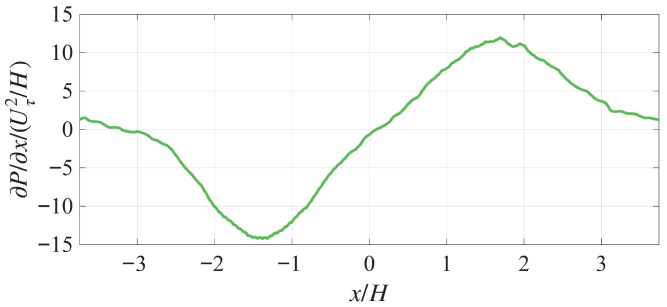
Profile of the mean streamwise pressure gradient from case FZA_30. Sampled 0.01H above the top plate of the APPS.

The symmetric insert along the channel centerline creates a streamwise transition from FPG to ZPG to APG (and then back to ZPG and FPG due to the streamwise periodic boundary condition) in the channel (shown in Figure 3), enabling investigation not only of the pore-flow response within the APPS under various local pressure gradients, but also of how the pore flow evolves in response to the spatial changes in these gradients. The insert is formed by trimming a NACA 0024 airfoil at its point of maximum thickness, mirroring the aft section to the front, and adding a flat segment between the diverging leading portion and the converging trailing end. The central flat region serves to smooth the transition between the FPG and APG, providing an opportunity to observe how the pore flow responds to a change in the sign of the pressure gradient. This insert is designed to produce a symmetric variation in the channel cross-section and thereby a quasi-symmetric FPG and APG along the *x* direction. As will be shown, no flow separation is observed over the insert, and the wake emanating from the trailing edge generates only weak vortex formation and shedding (i.e., minor changes to the turbulence). The entire insert is 4H long with a 1.212H-long flat section. Because the streamwise periodicity of the channel flow, the distance between the trailing edge of the insert and the leading edge of its downstream image is 3.489H. In this paper, the origin of coordinates is set to be at the middle of the insert on the channel centerline. Figure 4 exhibits the streamwise pressure gradient on the APPS produced by the insert.

A total of six cases were conducted, as summarized in Table 1. One case without the centerline insert, referred to as the ZPG case, is performed to assess the drag penalty of the APPS under canonical ZPG flow conditions. The other five cases include the insert and are therefore denoted as ‘FZA’, representing the FPG–ZPG–APG pressure-gradient transition along the channel. Case FZA_30 examines the full APPS subjected to the pressure gradients introduced by the insert. Cases FZA_Cyl (cylinder-only) and FZA_Plate (plate-only) remove either the top or bottom portion of the APPS, respectively, to demonstrate the necessity of both components. Case FZA_15 incorporates more inclined slots at 15° with respect to the horizontal direction to possibly accentuate the APG-preference of the wall-normal mass exchange. These cases with the channel insert are compared with Case FZA_SM (i.e., smooth), in which no surface structures are applied to the channel walls. The pore flow within the APPS due to the PGs, as well as the associated drag/thrust, are the essential quantities of concern for the simulations.

### 2.3. Numerical Methods

The DNS are conducted using a well-validated second-order finite difference code that solves the non-dimensional incompressible Navier-Stokes equations [31,37,38,39,40]:

(1) $$\frac{\partial u_{k}}{\partial x_{k}}\;=\;0;\;\;\frac{\partial u_{i}}{\partial t}+\frac{\partial u_{i}u_{k}}{\partial x_{k}}\;=\;-\frac{\partial p}{\partial x_{i}}+\frac{1}{Re}\frac{\partial^{2}u_{i}}{\partial x_{k}^{2}}+f_{i}.$$

The indices i,k=1, 2, and 3 represent the streamwise, wall-normal, and spanwise directions, respectively. The velocity components aligned with these directions are denoted by *u*, *v*, and *w*. The no-slip boundary condition on the surface of the APPS and the insert is enforced by an immersed boundary method based on the volume-of-fluid (VOF) approach through the term $f_{i}$ in Equation (1) [31,41]. Periodic boundary conditions are applied in the streamwise and spanwise directions. All cases use a grid with $N_{i}\times N_{j}\times N_{k}\;=\;2880\times 392\times 1440=1.6$ billion grid points. The grid is uniform in *x* and *z*. For the *y* grid, it is uniform from the wall to the top of the APPS, and then stretched toward the channel centerline using a hyperbolic tangent profile with less than 3% stretching. Note that the flow around the insert at the channel centerline is not the primary focus. The grid resolution there is chosen to adequately capture the overall flow deviation and the resultant pressure-gradient variation, rather than to resolve the fine flow details near the insert surface. The minimum surface radius of the curvature-to-grid spacing ratio is 25, and the grid size in *y* in the range of the insert is less than 0.015*H*. For the APPS, each cylinder is resolved with 12 grid points per diameter in the wall-parallel plane. 50 grid points resolve the APPS up to its top surface in the wall-normal direction, and each slit opening is resolved with 6 grid points across its width. Turbulence, as well as the flow within the APPS, is well resolved by these resolutions. The maximum grid spacing in wall units is $\Delta x^{+}=3.9$, $\Delta y_{1}^{+}=0.54$, and $\Delta z^{+}=3.52$. The grid resolution is comparable to or finer than the one used in [24,26,31] to resolve the flow at the sub-denticle scales, and in the DNS of turbulent channel flows [42,43,44]. Nevertheless, a grid convergence study (see Appendix A) is performed using this grid and another one that is refined by 25% in the wall-parallel directions. The wall-normal grid remains unchanged since the pore flow is mostly wall parallel, and our current resolution resolves it by 50 grid points (less than one wall unit). The results indicate that the pore flow and force statistics differ only slightly between the two resolutions. Therefore, the current resolution is sufficient to resolve the flow within the APPS and the turbulent channel flow. Each case is advanced to a statistically steady state and then continued for 10 $H/u_{\tau }$ to collect statistics. This is equivalent to about 28 flow-through times over the streamwise domain by the bulk flow. The statistics reported here remain unchanged when 75% of the snapshots are used for averaging. Mean quantities are averaged in both time and the spanwise direction within the fluid domain (intrinsic averaging) as well as between the top and bottom two halves of the channel. In the following, the primary flow variables are expressed in capital letters, and the fluctuations are denoted by ${\left(\right)}^{\prime }$.

### 2.4. Experiments

**Figure 5:**
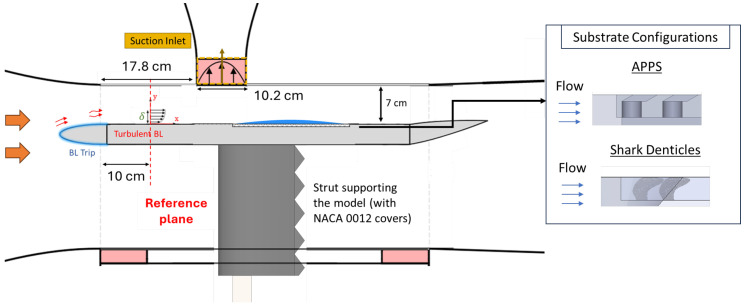
Schematic of the experimental setup in which suction at the upper wall induces an APG or separated flow over a ‘floating’ flat-plate model. Not to scale for clarity. The flow is from left to right. The APPS extend over the time-average separated flow and span the full model width, with the substrate configuration relative to the flow direction illustrated.

**Figure 6:**
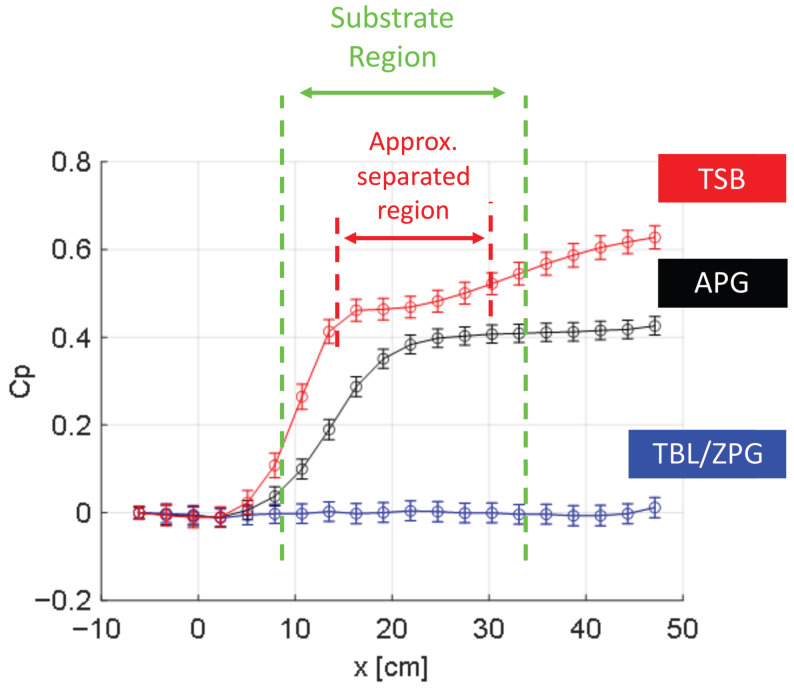
Pressure coefficient variation for the experimental conditions in Table 2 with the substrate region and the approximate separated bubble region (under TSB condition) highlighted. The TSB and TBL/ZPG data is also reported in [45].

Experiments are conducted on a flat-plate model in an open-loop, low-speed wind tunnel equipped with a vibration-isolated and acoustically-treated flow suction setup [45,46] depicted in Figure 5. The model consists of an elliptical nose cone with zig-zag extrusions that trip the turbulent boundary layer (TBL) and a trailing-edge flap adjusted to avoid leading-edge separation. An APG is induced over the flat plate model using a variable-speed suction fan. Tunnel and suction fan speeds are set to establish an APG or separated flow over the flat plate model. Incoming flow conditions are acquired in a reference plane (Figure 5) sufficiently downstream of the leading edge to allow the turbulence to reach equilibrium while far enough upstream not to be affected by flow suction. Table 2 summarizes the incoming TBL parameters under the three experimental conditions at the reference plane: TBL/ZPG, APG, and turbulent separating bubble (TSB), respectively. Figure 6 depicts the coefficient of pressure, $C_{p}=\Delta P/\left(\frac{1}{2}\rho U_{\infty }^{2}\right)$ (where $U_{\infty }$ is the freestream velocity at the reference plane), acquired using 28 HCLA pressure sensors on the centerline on the flat plate model [45]. The uncertainty analysis accounts for the error propagation associated with $C_{p}$ calculation.

An ATI Nano 25 load cell is attached to the base of the strut supporting the model for drag measurements, while ensuring the model is isolated from the wind tunnel walls [45]. The load cell’s *x*-axis is oriented in the streamwise direction for drag measurements. The total drag force acting on the flat plate and the supporting structure is obtained. The average value of the load cell zero offset measured before and after the run is used to account for any load cell drift during experiments. Drag coefficients are calculated for drag forces ($F_{D}$) acquired: $C_{D}=F_{D}/\left(\frac{1}{2}\rho U_{\infty }^{2}A_{\mathrm{fp}}\right)$, where $A_{\mathrm{fp}}$ is the planform area of the model. Several independent experiments are conducted for each flow condition. Uncertainty calculation includes load-cell uncertainty, pressure sensor/velocity measurement uncertainty, and standard error with a 95% confidence interval ($SE=1.96\sigma /\sqrt{n}$, where *n* is the number of independent measurements and σ is the standard deviation).

**Figure 7:**
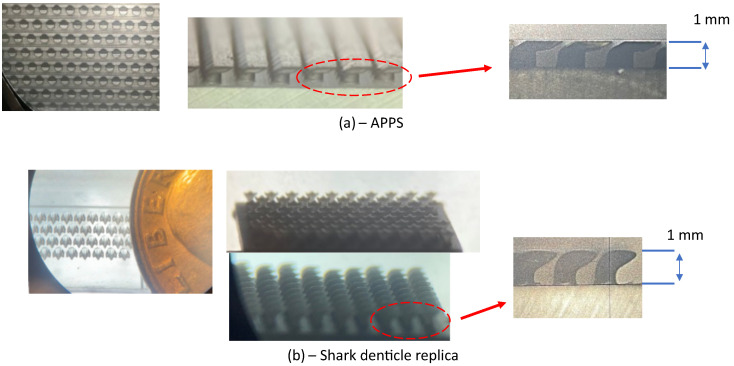
Microscopic and Image comparator images of the sample 3D prints of the (**a**) APPS and (**b**) Shark denticle replica using the 3D model in [31]. A US quarter is placed next to the denticle array in (**b**) for scale.

The APPS is designed to mimic the one used in the FZA_30 case described in Section 2.2.The physical and non-dimensionalized scales are listed in Table 3. Note that the length scales non-dimensionalized by the boundary layer thickness differ between simulations and experiments; however, those normalized by the viscous length scale are close because the experimental friction Reynolds number is substantially lower ($Re_{\tau }\approx 268$). As a result, the near-wall turbulence over the APPS is similar in both the numerical simulations and the experiments. Nevertheless, only qualitative comparisons can be made, since the inviscid pressure gradients imposed on the APPS are totally different between the two settings, so does the outer wall-bounded flows examined here. Stereolithography (SLA) 3D printing technique is selected to fabricate substrates due to the high-resolution small-scale features [47,48]; a FormLabs Form 4L MSLA 3D printer is used, which is equipped with an anti-aliasing filter for sub-pixel resolution and has a wall-parallel resolution of 46 μm and a wall-normal resolution as low as 10 μm [49]. The APPS is printed with a total feature height $\delta_{h}\;=\;1\;\mathrm{mm}$, which is limited by 3D printing constraints. In addition, a substrate of shark-denticle (SD) replica from ref. [31] is tested as well using the same feature height $\delta_{h}\;=\;1\;\mathrm{mm}$, where the denticle shape is derived from Isurus Oxyrinchus (Shortfin Mako) using the 3D model described in ref. [31]. The substrates span both upstream and downstream of the time-averaged separation bubble in the streamwise direction in the TSB case (the substrate region relative to the $C_{p}$ profile is highlighted in Figure 6) and extend to the edge of the model in the spanwise direction to account for the three-dimensionality of the separated flow. The substrates are flush-mounted to the flat plate model, such that the upper surface of the flat plate is aligned with the surface, with the slots in the top plate of the APPS (or the tail of the denticle crown) facing the downstream as depicted in Figure 5. Figure 7 depicts microscopic images of the APPS and shark denticle array fabricated and used in the experimental investigation; while the scale of the features is very small, the fine details are well produced with high accuracy using our 3D printing method. Geometric fidelity of the printed denticles and APPS samples is assessed using an optical comparator and microscopy. The largest measured surface variation is <20 μm, corresponding to $k^{+}\approx$1.15 based on the experimental friction velocity. Since $k^{+}$ < 5 characterizes the hydromechanically smooth regime, these variations are not expected to influence the flow. The printer’s anti-aliasing exposure provides sub-pixel lateral resolution, and microscopy shows no detectable geometric difference between 25 μm and 50 μm layer heights. The printed models, therefore, accurately reproduce the intended denticle and APPS geometry.

## 3. Results

### 3.1. Simulation Results: Instantaneous Flow

**Figure 8:**
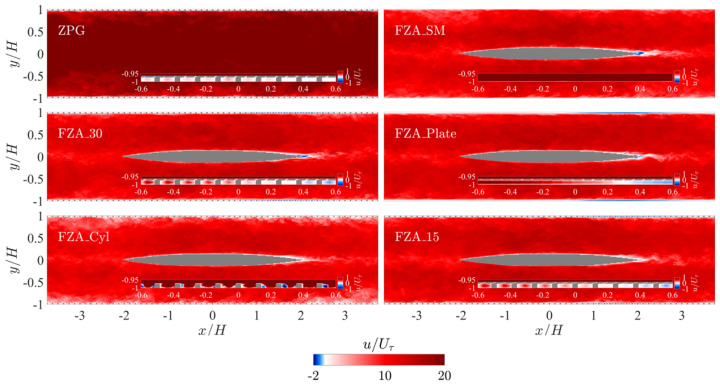
Contours of instantaneous streamwise velocity taken at the mid-span of the channel. Most of the region with x<0 is under FPG, while the region with x>0 experiences APG. The area near x=0, as well as the zones close to the periodic inflow and outflow boundaries, are quasi-ZPG transiting between the FPG and APG. The APPSs are applied on the top and bottom wall at y/H=±1. Insets show the region close to the wall/APPS near x=0. Note that the color bar is different for the main figure and the inset.

**Figure 9:**
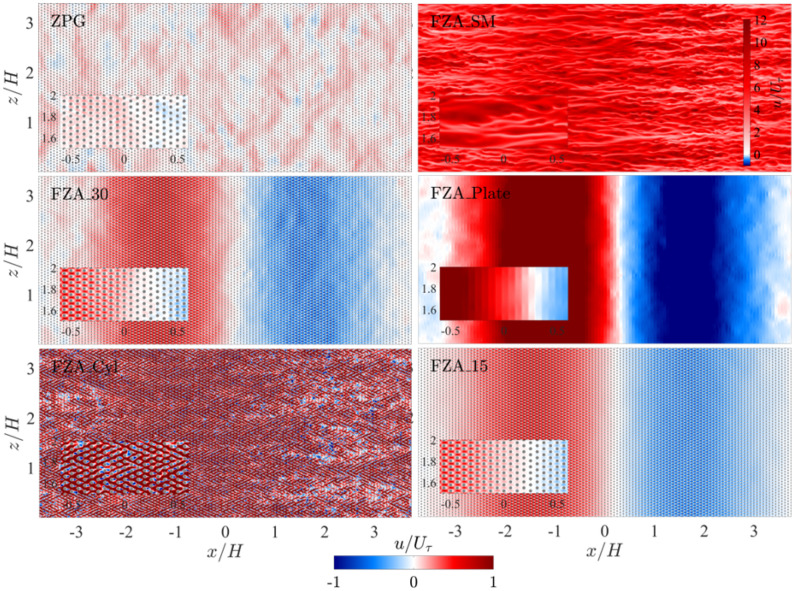
Contours of instantaneous streamwise velocity in the wall-parallel plane at the cylinder half height (y/H=−0.9875). Most of the region with x<0 is under FPG, while the region with x>0 experiences APG. The area near x=0, as well as the zones close to the periodic inflow and outflow boundaries, are quasi-ZPG transiting between the FPG and APG. Insets show the region near the wall/APPS at x=0. Note that the color bar for Case FZA_SM differs from that of the other cases.

Figure 8 and Figure 9 show the instantaneous streamwise velocity at the mid-span of the channel and the wall-parallel plane at the half cylinder height, respectively, for each case. In the ZPG case, both forward (in the positive-*x* direction as defined with respect to the bulk flow direction) and reverse (opposite to the bulk flow) pore flow regions appear intermittently but exhibit a recognizable spatial pattern. However, their spatial distribution, including the orientation and length scale of the coherence of these structures, does not seem to align with the typical turbulent structures observed in channel flow. In particular, no streamwise-elongated regions resembling those shown in the FZA_SM case are observed. The intermittent distribution suggests that the forward and reverse pore flows could largely cancel out over the wall-parallel plane.

The effects of the insert can be clearly observed in the other five cases. The flow accelerates due to the FPG and subsequently decelerates later over the converging trailing section. The insert generates a minor reverse flow region near its trailing edge without forming any large-scale vortices. Instead, it produces a reduced-velocity wake along the channel centerline. When no APPSs are present on the channel walls in case FZA_SM, the flow on the wall exhibits streamwise elongated streaks. The acceleration does not significantly relaminarize the flow with the present insert. Rather, it causes faster and wider streaks in the region of the insert. Downstream of this region, the APG reduces their streamwise coherence length.

In Cases FZA_30, FZA_Plate, and FZA_15, the pore flow is directed forward over the FPG half of the channel but reverses over the APG half. A spatial intermittent pattern similar to those observed in the ZPG or FZA_SM is absent, except in the regions that are approximately ZPG—namely, near x=0 and in the zones with |x|/H>3 between consecutive inserts formed by the streamwise periodic boundary conditions. The transition from forward to reverse flow in the middle-insert ZPG region does not coincide with the geometric middle point (x=0). Because of the gradual development of the APG, the acceleration and subsequent reversal of the forward flow extend some distance, resulting in a delay relative to the geometry. Across the three cases, we observe that FZA_Plate generates a strong pore-flow motion even in the absence of the bottom cylinder layer—contrary to our initial assumption that the cylinders were essential for initiating pore flow. This finding indicates that pore flow can occur without the cylinder array, and may in fact become stronger when the cylinders are removed. Nevertheless, in FZA_30 and FZA_15 we do observe a clear channeling effect, where the flow locally accelerates through the gaps between cylinders, as shown in the insets of Figure 9. In Section 3.3, we further examine the relationship between pore-flow speed and the generation of forces. We show that, although the presence of cylinders reduces the overall pore-flow velocity, the flow redistribution created by the channeling effect can increase the efficiency of thrust harvesting. In other words, pore-flow magnitude alone does not directly dictate thrust production; rather, the cylinder-induced modification of the flow plays a more consequential role.

Another notable feature of the pore flow in these three cases is its sensitivity to the inclination angle of the slots in the top layer. The 15° slots are expected to enhance the APG preference for inward flow penetration over the APPS, thereby producing a stronger reverse pore flow than the forward one under the FPG. However, our results show that this effect is minimal. No significant difference is observed in the instantaneous flow field between the forward and reverse pores. Comparing the 30°-slot cases (FZA_30 and FZA_Plate) with Case FZA_15, the most pronounced instantaneous difference lies in the pore flow spatial fluctuation patterns, particularly in the quasi-ZPG regions. These fluctuation patterns resemble those in the ZPG case rather than the characteristic turbulent streaks, and are more pronounced in Cases FZA_30 and FZA_Plate than in Case FZA_15. This indicates that more inclined slots are only effective at filtering out spatial inhomogeneities, resulting in more uniform pore flow.

Compared with the three cases that include a top plate, Case FZA_Cyl illustrates how removing the top layer affects flow behavior. In this configuration, the flow around the bottom cylinders can no longer be considered pore flow, as it is directly exposed to the main channel flow. As shown in Figure 8 and Figure 9 for Case FZA_Cyl, strong forward flow through the cylinder array dominates the cylinder region, generating local separation zones behind the cylinders, which are most pronounced in the APG region. No bulk reverse pore flow is observed. This result demonstrates that the top layer of the APPS is essential for generating reverse flow under an APG. Without the flow-isolating top layer, the cylinder array behaves merely as surface roughness rather than as a substrate. The cylinder height in wall units is 37.5, corresponding to transitional roughness.

### 3.2. Simulation Results: Mean Characteristics

**Figure 10:**
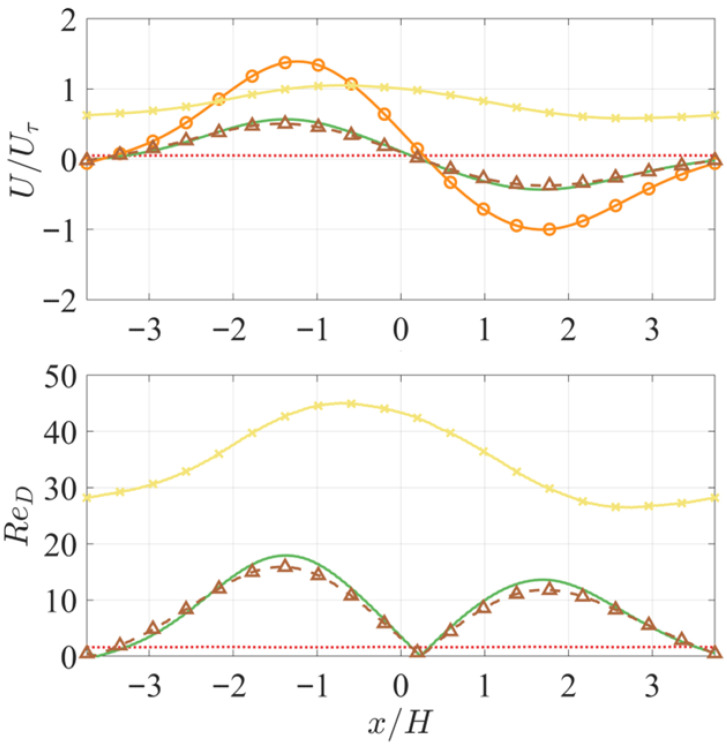
Profiles of the mean streamwise velocity in the cylinder region of the APPS and corresponding cylinder Reynolds number. 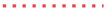 ZPG, 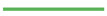 FZA_30, 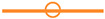 FZA_Plate, 
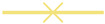
 FZA_Cyl, 
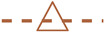
 FZA_15.

Figure 10 compares the time- and spanwise-averaged mean pore flow among the cases. The overall trends are consistent with the instantaneous flow fields, as the features described above remain valid across all time instants. The insert-induced pressure gradients are highly steady, resulting in pore flows that are modulated only by turbulent structures passing over the APPS. In the ZPG case, the mean pore flow velocity is 0.0525$U_{\tau }$, which is negligible when compared with either the channel bulk velocity in the same case (<$0.3\%U_{\mathrm{bulk}}$), or with the pore flow in other cases which peaks around 0.5 to 1.5$U_{\tau }$. Among all cases, FZA_Plate exhibits the highest mean pore flow magnitude, more than twice that of Cases FZA_30 and FZA_15. The latter two show nearly identical pore flow velocities in both the FPG and APG regions, with the FZA_15 case showing a slightly lower value. This indicates that no statistically significant enhancement in flow penetration through the APPS under APG relative to FPG was achieved by the more inclined slots, as initially hypothesized. Instead, it introduces a small additional resistance, without directional preference, possibly due to the longer passage length through the slots in the top plate. When the top plate is absent in Case FZA_Cyl, no mean reverse pore flow is observed across the entire domain.

The flow pattern behind the cylinders is characterized by the cylinder Reynolds number and the spacing [50,51,52]. $Re_{D}\;=\;UD/\nu$ with the mean velocity taken at the cylinder mid height is shown in Figure 10 as well. The peak $Re_{D}$ is 12-18 in Cases FZA_30 and FZA_15, and much greater at 26-45 when there is no top plate. Among others, Khalifa et al. [51] reported the wake pattern at various levels of porosity (i.e., spacing) and $Re_{D}$. For the cylinder array used in the current APPS, the porosity is 0.76. Khalifa et al. showed that for this porosity (Figure 20 in [51]), the pore flow between the cylinders is Darcian for $Re_{D}<1.15$, pore flow inertial effects become important for $Re_{D}>1.78$ and vortex shedding starts at $Re_{D}=47$. Therefore, in the mean sense, the pore flow in Cases FZA_15 and FZA_30 is in the regime where viscous forces are competing with inertia forces (Forchheimer regime), and local recirculation is present around each cylinder. When there is no top plate in Case FZA_Cyl, the inertia effect is augmented, thus vortex shedding may start to occur, plus the shedding of the shear layer from the exposed top of the cylinder, as shown in the instantaneous contours in Figure 8.

**Figure 11:**
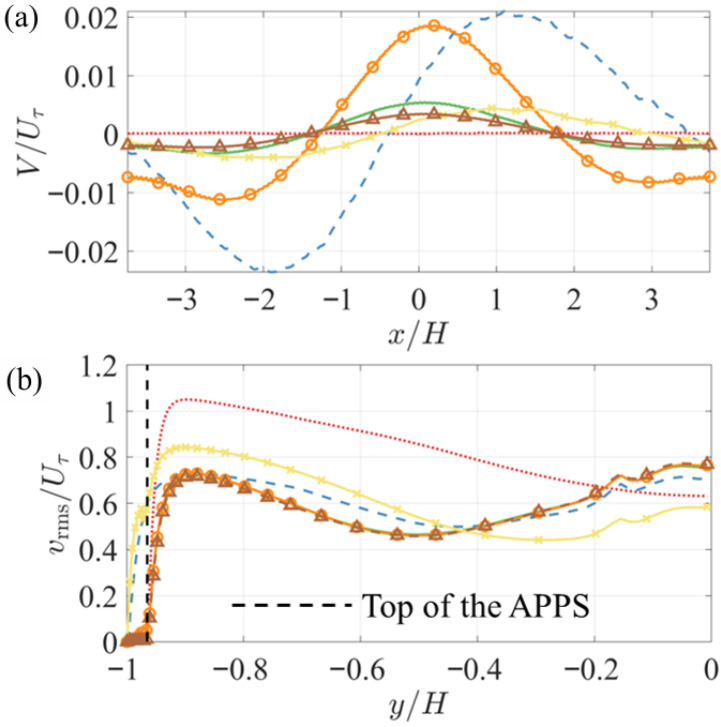
Profiles of (**a**) the mean wall-normal velocity through the top plate, measured at the mid-thickness of the top plate of the APPS, (**b**) the rms of the wall-normal velocity. 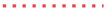 ZPG, 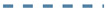 FZA_SM, 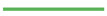 FZA_30, 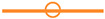 FZA_Plate,
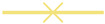
 FZA_Cyl, 
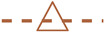
 FZA_15.

Comparing the FPG and APG sides, the peak forward pore flow velocity is greater than the peak reverse velocity. The ratio between the two is 1.38 in Case FZA_Plate and 1.32 in Cases FZA_30 and FZA_15. Since the pore flow originates from the channel flow above the top plate and is generated as fluid penetrates through it, mass conservation implies that a larger total downward flux into the APPS in Case FZA_Plate than in Cases FZA_30 and FZA_15. Moreover, when the pore flow transitions from forward to reverse in the quasi-ZPG region, it must re-enter the top plate through upward penetration, forming an outward flux through the top plate of the APPS. Figure 11a shows the wall-normal velocity through the top plate, measured at the mid-thickness of the plate. The profiles at the same wall distance in Cases ZPG and FZA_SM, where the APPS is absent, are also included for reference. The cases with the top plate (and thus a bulk mean pore flow) exhibit an inward (negative) flux in regions x/H<−1.35 and x/H>1.7, and an outward (positive) flux in between. This trend is qualitatively different from that in the ZPG, FZA_SM, and FZA_Cyl cases, where the flux follows the trend of the PG. Since the total inward flux must equal the total outward flux, the integrated volume flow rates across the two regions are necessarily the same. Therefore, since the outward transport is observed to be confined to a shorter streamwise distance (3.05H versus 4.44H), the outward velocities must be higher on average to carry the same total flux. This explains why the peak outward velocity is larger than the peak inward velocity: the inward motion is distributed more gradually, whereas the outward escape is concentrated spatially and thus faster. Among Cases FZA_Plate, FZA_30, and FZA_15, the peak inward velocity more than triples when the cylinder layer is removed. It should be noted that the mean wall-normal velocity plotted here is about two orders of magnitude smaller than $v_{\mathrm{rms}}$ near the top surface of the APPS (Figure 11b). Consequently, the motion into and out of the slots is not discernible in the instantaneous *v* contours in the wall-parallel domain. While the mean PG determines the overall direction of penetration, the near-wall turbulence primarily governs the instantaneous inhomogeneous penetration through the top plate. These observations indicate that the mass exchange between the main flow and the bottom-cylinder region, while shielded by the top plate, occurs as a mild, continuous process over the areas subjected to PGs, rather than as narrowly localized jets.

### 3.3. Simulation Results: Forces

The results discussed above show that the pore flow attains its maximum strength when the bottom cylinder layer is absent. It may be anticipated that the largest net force would also be generated under these conditions. However, in Case FZA_Plate, the fast pore flow can only create skin friction on the flat bottom surface of the APPS plate and the wall. In contrast, when the bottom cylinder array is present, both form drag and the additional skin friction over the cylinders’ side surfaces contribute to the total force.

**Figure 12:**
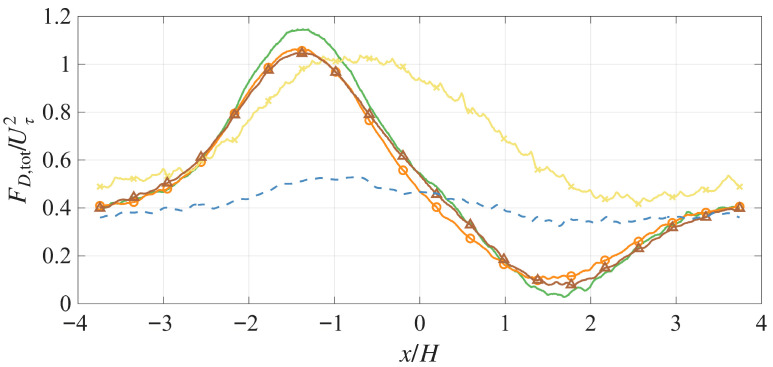
Profiles of the total force created by the APPS and the channel wall. 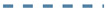 FZA_SM, 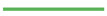 FZA_30, 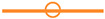 FZA_Plate, 
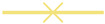
 FZA_Cyl, 
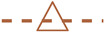
 FZA_15.

Figure 12 presents the total force generated in each case by the APPS and the channel wall. Note that the force generated by the APPS is obtained directly through the immersed boundary module of the solver whenever a structure is present on the wall, integrating the $-f_{i}$ term in Equation (1) in *y* up to the top of the APPS. This force represents the combined contribution of skin friction and pressure acting on the solid structure imposed by the immersed boundary. Because both components contribute to thrust or drag, separating them does not offer additional insight for the purposes of the present study. However, such decomposition may be valuable in future work aimed at optimizing thrust generation. The skin friction on the remaining channel wall surfaces between the cylinders is then added to this force to obtain the total force produced by the APPS. The only case in which wall drag is independently evaluated is the FZA_SM configuration, whose skin friction is used as a reference for quantifying changes in force.A ‘thrust’ is defined here as a reduction in the net downstream drag force exerted on the structure. While the top surface of the APPS remains exposed to friction from the outer flow (thus still producing drag), the bottom layer generates an upstream-directed force that contributes to drag reduction. If the reverse pore flow is sufficiently strong, the total force can become negative, indicating that the structure is not only free from downstream drag but is actually pushed upstream by the pore flow. The highest thrust (under APG) and drag (under FPG) are produced by the complete APPS in Case FZA_30. In Case FZA_Plate, despite the high velocity beneath the top plate of the APPS, the resultant force is comparable to that in Case FZA_15. Without the top plate in Case FZA_Cyl, the cylinder array alone generates a substantial drag penalty across the entire domain.

These results demonstrate that while the magnitude of the pore flow (i.e., flow rate) is important, its effectiveness in generating force also depends on the underlying mechanism of force production. To maximize thrust generation under APG, a bottom cylinder array is more advantageous than a void layer beneath the top plate. It is also important to note that the force generated by the lower region of the APPS corresponds to the drag that is typically undesirable in conventional porous media applications. Its reversal in direction under an APG, however, makes it beneficial in certain applications. A higher thrust, though advantageous, also implies that a lower pore flow rate under the same APG. Therefore, optimizing the APPS for a given APG to achieve maximum thrust harvesting requires optimization between the flow rate and the force generation.

### 3.4. Experiment Results

**Figure 13:**
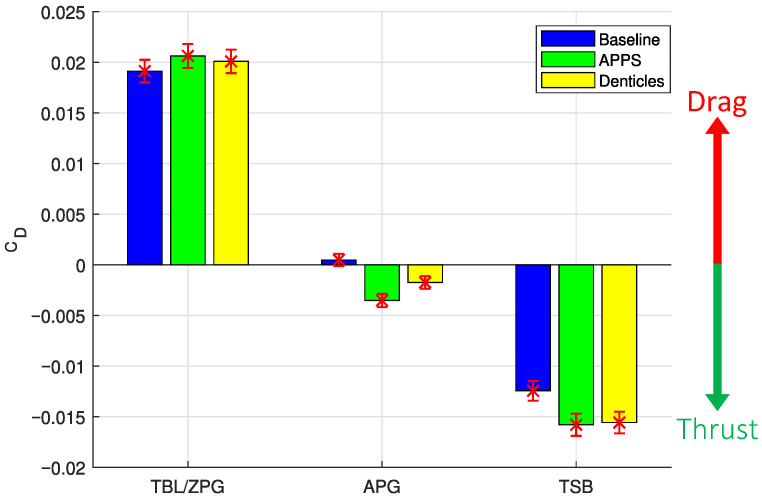
Drag coefficient ($C_{D}$) measured from experimental analysis under the three flow conditions detailed in Table 2. Error bars include equipment uncertainty and standard error as detailed in Section 2.4.

Figure 13 and Table 4 illustrates experimental $C_{D}$ under TBL/ZPG, APG, and TSB flow conditions for the flat plate (without any structures) and with the APPS and SD substrate attached. Repeatability is confirmed for all cases with multiple experimental runs, with deviations <2% in forces measured across runs. A slight increase in drag is observed with the APPS and SD under ZPG TBL conditions. The flat plate drag coefficients measured under APG conditions are lower than the ZPG as the flow decelerates, and a mild drag reduction is observed when the APPS and SD substrates are attached. Comparing the two substrates, the APPS has a larger drag reduction. When the APG is stronger and leads to flow separation under the TSB flow conditions, a higher drag reduction is observed, again with the APPS relative to the SD, suggesting more efficient reverse-flow enhancement/thrust with the APPS. Note that under the TSB condition, the flat plate also produces a thrust, which we interpret as a result of the relatively high-pressure region generated behind the flap. Both APPS and SD lead to an increase in thrust/drag reduction under TSB conditions relative to the flat plate. This additional drag reduction of approximately 27% is significant and aligns with numerical results for separated flow in ref. [31]. This further validates that the current APPS configuration is effectively reproducing the performance of the SD substrate and even exceeds it in drag reduction.

## 4. Discussion and Conclusions

Direct numerical simulations and experiments are conducted to examine the drag-reduction performance of an Anisotropic Permeable Propulsive Substrate (APPS) inspired by shark denticles. The APPS consists of a top plate with cut-through slots and a bottom array of vertical cylinders. The top plate mimics the crown of shark denticles, which shield the pore flow from the outer flow, while allowing vertical mass exchange. The bottom layer enables the enhancement of a reverse pore flow under APG that can generate thrust.

The numerical simulations investigate how the pore flow responds to variations in pressure gradient and substrate geometry, because these factors fundamentally determine thrust generation. The APPS demonstrates high effectiveness under APG, generating a robust reverse pore flow and achieving meaningful drag reduction consistent with its biomimetic design intent. This performance highlights that the core mechanism of the system functions as intended and that the structure successfully leverages APG conditions to reduce drag. The backward-inclined slots on the top plate, however, do not exhibit the preferential APG response we initially anticipated. While their geometry suggested that APG-driven flow might more readily enter the slots, the simulations show no significant enhancement in slot-mediated pore flow under APG compared with FPG, indicating that this secondary feature did not perform as hypothesized. The direction and magnitude of the pore flow and the resultant force were shown to depend primarily on the local pressure gradient over the APPS, consistent with the canonical Darcy–Forchheimer behavior of porous media. Overall, the APPS exhibits two key advantages over conventional porous media. First, the flat top plate isolates the underlying porous region from the outer flow, so that under ZPG—where no pore flow is expected in the absence of a driving pressure gradient—the external flow encounters an effectively smooth surface rather than a rough, exposed porous substrate. This prevents the additional drag that conventional porous media would introduce under such conditions. Second, when an APG is present, the same isolating top layer constrains the pore flow within the lower porous region to move predominantly in the wall-parallel direction by suppressing its wall-normal escape or relief. This confinement forces the pore flow to act along the streamwise direction, thereby enabling the generation of upstream thrust. Experimental measurements confirm that the APPS achieves comparable (or, in some cases, improved) drag-reduction performance relative to shark denticles.

Ongoing work focuses on enabling unidirectional (reverse-only) APG-favorable pore flow, optimizing the geometry for maximum thrust generation, and visualizing the flow field with PIV/PTV. Future work may focus on understanding the effects of unsteady pressure gradients to better capture realistic boundary-layer dynamics, as well as making the APPS design ready for practical implementation by following design frameworks such as the one proposed in [53].

## Figures and Tables

**Table 1:** Summary of cases. Five cases feature an insert enabling the FPG–ZPG–APG (FZA) transition. The slot inclination angle is 30° unless otherwise indicated. The schematic is not to scale for clarity.

Case Name	Channel Insert	Features	Configuration Schematic
ZPG	No	Full APPS	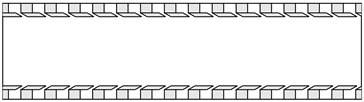
FZA_SM	Yes	No APPS	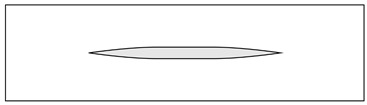
FZA_30	Yes	Full APPS	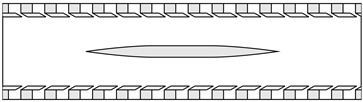
FZA_Cyl	Yes	Cylinder only	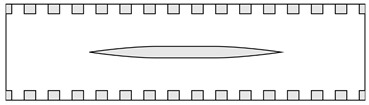
FZA_Plate	Yes	Top plate only	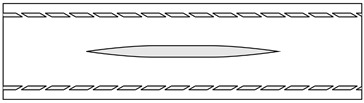
FZA_15	Yes	Full APPS, 15° slots	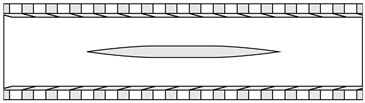

**Table 2:** TBL conditions at the reference plane (Figure 5).

	$U_{\infty }$ (m/s)	δ (mm)	Reθ	Shape Factor
TBL	14.7	6.99	782	1.62
APG	14.8	6.99	810	1.60
TSB	13.7	6.93	762	1.68

**Table 3:** Geometry parameters of the APPS used in the experiments (and numerical simulations, if applicable). $\delta_{\nu }$ is the viscous length scale. The inclination angle of the slots is 30° for the APPS 3D printed in the experiments.

	mm	Normalized by δ(H)	Normalized by $\delta_{\nu }$
Total height	1.00	0.143 (0.035)	38.2 (52.5)
Cylinder height	0.714	0.102 (0.025)	27.3 (37.5)
Cylinder diameter	0.857	0.122 (0.030)	32.8 (45.0)
Top plate thickness	0.286	0.0409 (0.010)	10.9 (15.0)
Slot width	0.400	0.0571 (0.014)	15.3 (21.0)
Slot spacing	1.78	0.254 (0.0484)	68.0 (93.6)

**Table 4:** Drag coefficient values ($10^{2}C_{D}$) for each flow condition with flat plate, APPS, and denticle substrate depicted in Figure 13. A negative drag coefficient corresponds to a net upstream force (thrust).

Case	TBL	APG	TSB
Flat Plate	1.91±0.11	0.5±0.06	−1.24±0.10
APPS	2.06±0.12	−0.35±0.06	−1.58±0.11
Shark Denticles (SD)	2.01±0.12	−0.17±0.06	−1.56±0.11

**Figure A1:**
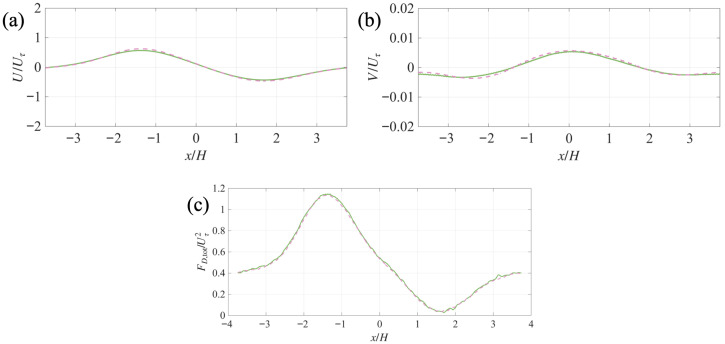
Comparisons of (**a**) the mean streamwise velocity at the half-cylinder height (refer to Figure 10a); (**b**) the mean wall-normal velocity through the top plate (refer to Figure 11a); and (**c**) the total force created by the APPS and the channel wall. (refer to Figure 12). 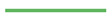 coarser grid, 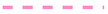 finer grid.

## Data Availability

The raw data supporting the conclusions of this article will be made available by the authors on request.

## Nomenclature

δ	Boundary layer thickness
$\delta_{h}$	APPS feature height
$\delta_{w}$	Denticle width (following [31])
$\delta_{x}$	Streamwise spacing of cylinder array
$\delta_{z}$	Spanwise spacing of cylinder array
ν	Kinematic viscosity
θ	Momentum thickness
$A_{\mathrm{fp}}$	Reference planform area of the flat-plate model used in the drag coefficient definition
$C_{D}$	Drag coefficient, $C_{D}=F_{D}/\left(\frac{1}{2}\rho U_{\infty }^{2}A_{fp}\right)$
$C_{p}$	Pressure coefficient, $\Delta P/(\frac{1}{2}\rho U_{\infty }^{2})$
*D*	Cylinder diameter
$F_{D,\mathrm{tot}}$	Total streamwise force created by the APPS and the channel wall
*H*	Half-channel height
$k^{+}$	3D printing surface variation height in viscous units
$Re_{\tau }$	Friction Reynolds number, $Re_{\tau }\;=\;U_{\tau }H/\nu$
$Re_{\theta }$	Momentum-thickness Reynolds number, $Re_{\theta }\;=\;U_{\infty }\theta /\nu$
$Re_{D}$	Reynolds number based on cylinder diameter, $Re_{D}\;=\;UD/\nu$
*U*	Intrinsic-averaged streamwise velocity
*u*	Instantaneous streamwise velocity
$U_{\infty }$	Freestream velocity
$U_{\tau }$	Friction velocity
*V*	Intrinsic-averaged wall-normal velocity
$v_{\mathrm{rms}}$	Root-mean-square of the wall-normal velocity fluctuation
*x*	Streamwise direction
*y*	Wall-normal direction
*z*	Spanwise direction

## Appendix A. Grid Refinement Study

Grid convergence of the DNS is verified by comparing results from two grid resolutions for case FZA_30. The coarser grid is the one reported in this study. A finer one, featuring 3600 grid points in the streamwise direction (25% finer), 392 in the wall-normal direction (unchanged), and 1800 in the spanwise direction (25% finer), is also used, which increases the total number of grid points to 2.54 billion. In this finer grid, only the wall-parallel directions are refined because the wall-normal resolution across the APPS already meets DNS requirements ($\Delta y^{+}<1$) throughout the APPS region, and the pore flow exhibits minimal wall-normal variation due to the geometric confinement imposed by the top plate. All quantities of interest show clear grid convergence between the two resolutions. Figure A1 demonstrates the excellent agreement between the two grids in the mean pore flow velocity, transpiration (wall-normal) velocity through the top plate of the APPS, and the integrated streamwise force over the APPS. Therefore, due to the close agreement between the two resolutions and the substantially higher computational cost of the finer grid, the coarser grid is used for all remaining cases.
